# Modulation of Airway Expression of the Host Bactericidal Enzyme, sPLA2-IIA, by Bacterial Toxins

**DOI:** 10.3390/toxins15070440

**Published:** 2023-07-03

**Authors:** Yongzheng Wu, Erwan Pernet, Lhousseine Touqui

**Affiliations:** 1 Unité de Biologie Cellulaire de l’Infection Microbionne, CNRS UMR3691, Institut Pasteur, Université de Paris Cité, 75015 Paris, France; yongzheng.wu@pasteur.fr; 2 Groupe de Recherche en Signalisation Cellulaire, Département de Biologie Médicale, Université du Québec à Trois-Rivières, Trois-Rivières, QC G8Z 4M3, Canada; 3 Sorbonne Université, Inserm U938, Centre de Recherche Saint-Antoine (CRSA), 75012 Paris, France; 4 Institut Pasteur, Université de Paris Cité, Mucoviscidose et Bronchopathies Chroniques, 75015 Paris, France

**Keywords:** bacterial toxins, sPLA2, host immunity, inflammation

## Abstract

Host molecules with antimicrobial properties belong to a large family of mediators including type-IIA secreted phospholipase A2 (sPLA2-IIA). The latter is a potent bactericidal agent with high selectivity against Gram-positive bacteria, but it may also play a role in modulating the host inflammatory response. However, several pathogen-associated molecular patterns (PAMPs) or toxins produced by pathogenic bacteria can modulate the levels of sPLA2-IIA by either inducing or inhibiting its expression in host cells. Thus, the final sPLA2-IIA concentration during the infection process is determined by the orchestration between the levels of toxins that stimulate and those that downregulate the expression of this enzyme. The stimulation of sPLA2-IIA expression is a process that participates in the clearance of invading bacteria, while inhibition of this expression highlights a mechanism by which certain bacteria can subvert the immune response and invade the host. Here, we will review the major functions of sPLA2-IIA in the airways and the role of bacterial toxins in modulating the expression of this enzyme. We will also summarize the major mechanisms involved in this modulation and the potential consequences for the pulmonary host response to bacterial infection.

## 1. Role of sPLA2-IIA in Infectious and Inflammatory Diseases

### 1.1. General Biological Functions of sPLA2-IIA

Phospholipase A2 (PLA2) enzymes hydrolyze the sn-2 position of phospholipids, resulting in the production of free fatty acids and lyso-phospholipids [1,2]. These enzymes are classified into two major families: the low molecular weight-secreted PLA2 (sPLA2) and the high molecular weight intracellular PLA2, such as the cytosolic PLA2 (cPLA2) [1,2]. Based on the number and position of their disulfide bridges, sPLA2 can be classified into several different types, one of which is sPLA2-IIA. PLA2 have been shown to release free arachidonic acid (AA), the precursor of proinflammatory eicosanoids, and to bind to specific receptors present on host surface membranes [3,4]. Initially, sPLA2-IIA was suggested to play a role in the development of various inflammatory diseases [5,6]. For example, this enzyme can hydrolyze pulmonary surfactant phospholipids involved in acute respiratory distress syndrome (ARDS). In addition, sPLA2-IIA has been shown to induce neuronal apoptosis in ischemic stroke [7]. Other studies have also shown the involvement of sPLA2-IIA in atherosclerotic lesions [8,9,10], the hydrolysis of mitochondrial membranes released by platelets [11] and plasma lipoproteins [12]. sPLA2-IIA has also been shown to generate lipid mediators from membrane vesicles of platelets and erythrocytes [13].

Thus, it is clear that sPLA2-IIA can be involved in various pathophysiological processes, but its high bactericidal property (especially against Gram-positive bacteria) is now accepted as its most established biological role [5,6,14]. Therefore, it is important to explore the mechanisms by which bacterial toxins can modulate the expression of this enzyme and the pathophysiological consequences of this modulation.

### 1.2. PAMPs, Toxins and Innate Immune Response to Bacterial Infections

Pathogen-associated molecular patterns (PAMPs) are microbial motifs that are highly conserved across a wide range of pathogens. They are essential for the survival of these pathogens and their detection by host cells [15]. They include several virulence factors, such as lipopeptides, lipoteichoic acid (LTA) or peptidoglycans (PGN) of Gram-positive bacteria and lipopolysaccharides (LPS), pili or flagellin of Gram-negative bacteria as well as the double-stranded RNA (dsRNA) of certain viruses [16]. Recognition of PAMPs by specialized host cells is the first step in the host immune response, leading to an inflammatory response and elimination of the invading pathogens. This process involves the interactions of PAMPs with cellular receptors called ‘pattern recognition receptors’, or PRRs.

On the other hand, bacteria also produce a variety of toxins in response to various environmental challenges. These include exotoxins that are actively expressed and secreted into the extracellular media or injected into host cells during the infection process [17,18]. The interaction of pathogens with host cells initiates signaling processes that lead to the production of anti-microbial peptides (AMPs) by these cells. AMPs represent a large family of peptides ranging from 10 to 150 amino acids. In particular, 153 AMPs have been found in humans with net positive charges on the surface of the molecules [19], including defensins, cathelicidin, the type IIA secreted phospholipase A2 (sPLA2-IIA), etc. These antimicrobial molecules interact with bacteria to inhibit the synthesis of bacterial membrane phospholipids, cleave polysaccharides of the bacterial cell wall or increase the permeability of the bacterial membrane [19], which ultimately results in the eradication of the pathogens or the reduction of their proliferation. In particular, sPLA2-IIA has a high net positive charge of +17 [20]. Table 1 shows the reported effects of some PAMPs and toxins on sPLA2-IIA expression by host cells (Table 1).

### 1.3. The sPLA2-IIA, an Endogenous Antibiotic-Like Protein of the Host

The bactericidal activity of sPLA2-IIA is related to its ability to efficiently penetrate the cell wall of Gram-positive bacteria. This particular property is due to the high net positive charge of sPLA2-IIA (+17) [20], whereas Gram-positive bacteria have high net anionic charges due to the presence of the D-alanyl moiety covalently linked to lipoteichoic acid (LTA) [38], which is a major membrane component of Gram-positive bacteria. Thus, the highly efficient and rapid binding of sPLA2-IIA to LTA by electrostatic interaction promotes the penetration of sPLA2-IIA into the peptidoglycan layer, another major wall component of Gram-positive bacteria. This leads to efficient hydrolysis of bacterial membrane lipids and subsequent bacterial killing [14]. One study has reported a classification of mouse and human sPLA2 based on their ability to kill the Gram-positive bacterium *Staphylococcus aureus* [39] and showed that sPLA2-IIA is the most bactericidal sPLA2 type. Indeed, the concentration of sPLA2-IIA in human tears of healthy subjects exceeds 30 µg/mL, and only 15–80 ng/mL of this protein is sufficient to kill *S. aureus* [40]. Additionally, concentrations of sPLA2-IIA rapidly increase in host biological fluids as a result of inflammation or bacterial infection [4,41,42], as discussed in Part 4 of this section. These concentrations are virtually sufficient to kill all Gram-positive bacteria that may invade the host. Thus, sPLA2-IIA can be considered a major player in the host’s innate immunity.

### 1.4. Role of the sPLA2-IIA in the Gut Microbiota–Lung Axis

Given its potent and selective antibacterial activity against Gram-positive bacteria, it is tempting to speculate that sPLA2-IIA might be involved in shaping the gut and pulmonary microbiota [43,44]. Indeed, two recent studies have investigated the influence of sPLA2-IIA on the gut microbiota [43,44]. Using gain- or loss-function assays, sPLA2-IIA was shown to play a central role in the composition of the gut microbiota by reducing the proportion of Gram-positive strains [43,44]. Most importantly, sPLA2-IIA-driven changes in the gut microbiota contributed to alterations in local and extra-intestinal immune responses, leading to increased susceptibility to cancer and arthritis [43,44]. This evidence suggests that intestinal sPLA2-IIA has profound effects on the host immune response through modulation of the gut microbiota and it may also have an impact on the pulmonary immune response by influencing the airway microbiota. Interestingly, there is a privileged relationship and communication between the lung and the gut (known as the gut–lung axis) that is mediated by the microbiota [45], and the gut microbiota has been associated with immunity to viral and bacterial infections [45,46,47,48]. Therefore, future studies are needed to better address the specific role of sPLA2-IIA in microbiota changes and the associated effects on the gut microbiota–lung axis.

### 1.5. sPLA2-IIA Levels in Biological Fluids of Infectious and Inflammatory Diseases

sPLA2-IIA was originally identified in synovial fluid from patients with rheumatoid arthritis [49], suggesting its involvement in excessive inflammatory conditions, such as autoimmunity. Subsequent studies have reported elevated levels of sPLA2-IIA in biological fluids from inflammatory diseases, including ARDS [50], pancreatitis, sepsis, cardiovascular disease [4,41,42] and in nasal fluids from patients with allergic rhinitis [51]. ARDS is defined as a life-threatening lung injury characterized by non-cardiogenic pulmonary edema and arterial hypoxemia [50]. The alteration of pulmonary surfactant is a hallmark of ARDS, accounting for increased surface tension at the air–liquid interface, resulting in impaired gas exchange and alveolar collapse. We have shown that sPLA2-IIA hydrolyzes surfactant phospholipids and its expression is inhibited by the surfactant protein A [4].

On the other hand, the pioneering studies of the Weiss group have reported the presence of sPLA2-IIA in rabbit peritoneal exudates [42] and baboon plasma [52] at levels sufficient to kill *S. aureus*. They showed that pre-treatments of these fluids with a specific sPLA2-IIA neutralizing antibody abolished the killing activity of these fluids [42,52]. We have also identified potent bactericidal activity against *B. anthracis* (the causative agent of anthrax) in human bronchoalveolar lavage fluids (BALFs) from patients with ARDS [53]. This bactericidal activity was positively correlated with the levels of sPLA2-IIA in the BALFs, and pretreatment of the BALFs with the sPLA2-IIA inhibitor LY311727 abolished the bactericidal activity [53]. We also showed that alveolar macrophages (AMs), the major source of sPLA2-IIA in a guinea pig model of ARDS [22], released sufficient amounts of sPLA2-IIA to kill *B. anthracis* [53]. This killing activity was inhibited by pretreatment of the AM medium with LY311727, suggesting that sPLA2-IIA is the major anthracidal factor released by AMs [53].

## 2. Bacterial Toxins That Upregulate sPLA2-IIA Expression

**Figure 1:**
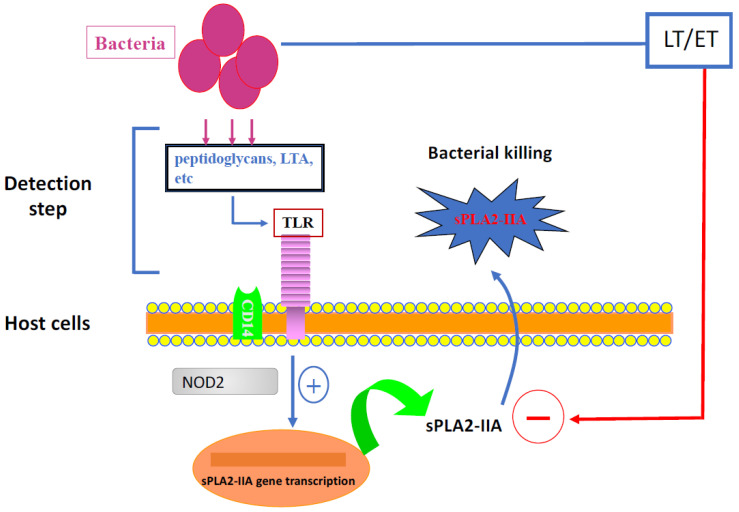
Positive vs. negative modulation of sPLA2-IIA expression in host cells by the *B. anthracis* toxins. At the early step of host cell infection by *B. anthracis*, this bacterium induces sPLA2-IIA expression and secretion by these cells via the action of various PAMPs, including peptidoglycan (PG) or LTA. This expression occurs via a NOD-dependent pathway. The enzyme sPLA2-IIA, once secreted in the medium, interacts with bacteria, leading to their killing. In parallel, *B. anthracis* produces the toxins ET and LT that downregulate sPLA2-IIA expression. The final concentration of this enzyme in the cell medium is the balance between the inducing (by PG) and the inhibiting effects of PLA2-IIA expression (by ET and LT).

The toxins produced by invading bacteria can modulate sPLA2-IIA levels, either inducing or inhibiting its expression by host cells. Thus, the resulting sPLA2-IIA concentration during the infection process would depend on the balance between the levels of toxins that stimulate and those that downregulate the expression of this enzyme. The synthesis and/or secretion of bacterial toxins will be activated once the bacteria come into contact with host cells. The inhibition of sPLA2-IIA expression by bacterial toxins highlights a mechanism by which certain bacteria can subvert the host immune system. However, the same bacterium can either induce or inhibit sPLA2-IIA production depending on the expression kinetics of the bacterial toxins involved in sPLA2-IIA modulation. In the following sections, we will summarize the most important studies in the literature that have investigated the effects of bacterial toxins on sPLA2-IIA expression in the respiratory system and their pathophysiological consequences (Figure 1).

### 2.1. LPS Is the Major Bacterial Toxin Inducing sPLA2-IIA Expression by Host Cells

It is widely accepted that LPS is the major initiator of the inflammatory response that is caused by Gram-negative bacteria [15]. This response involves a range of enzymes and mediators produced by host cells in response to stimulation by LPS. sPLA2-IIA is produced by host cells during the inflammatory response. The stimulation of sPLA2-IIA synthesis in the context of infectious diseases has mostly been attributed to bacterial toxins, such as LPS. LPS is a potent inducer of sPLA2-IIA expression in various rodent models of inflammatory diseases [54]. LPS from *E. Coli*, *P. aeruginosa* and *N. meningitidis* has been shown to induce sPLA2-IIA production from a variety of cells, including AMs and bronchial epithelial cells (BECs) [5,31,55]. However, LPS-induced sPLA2-IIA expression in human BECs is much less potent than in gpAMs [22,55]. Other studies reported that AMs isolated from ARDS patients did not respond to LPS to produce PLA2 [56]. On the contrary, the production of sPLA2-IIA was induced in control patients only after LPS treatment. Thus, PLA2 isoforms may serve as markers of immune status in ARDS [56].

LPS-induced sPLA2-IIA expression has been shown to be cell-specific and species-dependent. We have reported that LPS was able to induce sPLA2-IIA expression in AMs, the major source of this enzyme in a guinea pig model of ARDS [4,57]. In this model, the induction of LPS-induced sPLA2-IIA expression occurs via an autocrine/paracrine process dependent on TNFα. In baboons, TNFα also plays a role as an intermediate in LPS-induced sPLA2-IIA production [52]. These findings contrast with those in rat astrocytes and human hepatoma cells, where LPS-induced sPLA2-IIA expression occurred via a TNFα-independent process [58,59].

sPLA2-IIA has clearly been shown to play a role in the hydrolysis of dipalmitoyl-phosphatidylcholine (DPPC) in an animal model of LPS-induced ARDS [4,57,60]. DPPC is one of the major phospholipids of pulmonary surfactants. Its hydrolysis is associated with the loss of surface tension of the surfactant complex, leading to subsequent alveolar collapse, a key clinical feature of ARDS [4]. Another study reported that pretreatment with the sPLA2-IIA inhibitor S-5920/LY315920Na attenuated lung injury induced by oleic acid [60]. This effect was accompanied by protection against lung surfactant degradation and the associated production of the inflammatory lipid mediators thromboxane A2 and leukotriene B4 [60].

Subsequent studies showed that Azithromycin exerted anti-inflammatory properties on lung epithelial cells by inhibiting LPS-induced sPLA2-IIA expression, but had no effect on AMs [61]. On the other hand, treatment of brain microvascular endothelial cells (BMVECs) with LPS resulted in increased release of sPLA2-IIA and nitrite into the culture medium [33]. This release was decreased by pretreatment of the cells with an NO donor, sodium nitroprusside, suggesting that sPLA2-IIA expression is under the control of the NO–JAK3–STAT1 pathway [62]. Furthermore, sPLA2-IIA stimulates neuronal cell death via apoptosis [7], which appears to be associated with the production of arachidonic acid (AA) metabolites, particularly prostaglandin D2 (PGD2). In addition, sPLA2-IIA is involved in neurodegeneration in the ischemic brain, highlighting the potential therapeutic use of sPLA2-IIA inhibitors in stroke therapy [7].

### 2.2. Role of Pili in the Induction of sPLA2-IIA Expression in the Airways

In our previous studies, we investigated whether *N. meningitidis* toxins or PAMPs, other than LPS, were able to stimulate sPLA2-IIA expression using gpAMs [31]. This Gram-negative bacterium is the causative agent of meningococcal disease and is exclusively adapted to humans, with the nasopharynx being its natural habitat [63]. The results showed that *N. meningitidis* stimulates sPLA2-IIA synthesis through gpAMs and that pili mediate this stimulation [31]. Indeed, an LPS-deficient mutant of this bacterium was still able to induce sPLA2-IIA synthesis and a pili-deficient mutant showed a significantly lower ability to stimulate sPLA2-IIA expression than the wild-type strain. Moreover, a pili preparation isolated from an LPS-deficient *N. meningitidis* strain stimulated sPLA2-IIA production via a process involving NF-κB activation [31]. These studies demonstrated that pili, in addition to LPS, can induce sPLA2-IIA expression by *N. meningitidis.* However, the receptor(s) and the signaling pathways involved in pili-induced sPLA2-IIA expression were not investigated in this study. However, it should be emphasized that *N. meningitidis* has been shown to exert toxic effects on human epithelial and endothelial cells due to a synergistic effect of LPS and pili [64].

### 2.3. The Type 3 Secretion System (T3SS) Toxin, Exotoxin S (ExoS), Plays a Key Role in sPLA2-IIA Expression in the Airways

We have also examined the expression and role of sPLA2-IIA in the airways of patients with cystic fibrosis (CF) and showed that the *P. aeruginosa* PAK strain, but not PAMPs, isolated from this bacterium, including LPS, HSL, CpG, flagellin and pili, stimulated sPLA2-IIA synthesis by BECs from these patients [23]. This observation suggests that LPS-induced sPLA2-IIA synthesis is cell type dependent, and also prompted us to identify the *P. aeruginosa* virulence factors involved in sPLA2-IIA production by BECs in the context of CF. Compared to its parental strain, the PAK mutant lacking T3SS induced sPLA2-IIA expression at much lower levels, whereas the T2SS-deficient strain induced it at similar levels [23], suggesting a specific ability of T3SS to induce sPLA2-IIA expression. Moreover, using a pharmacological approach, we showed that the signaling pathways (NF-κB, AP-1 and MAPK), known to induce sPLA2-IIA expression in various cell systems (see ref [5]), did not play a role in ExoS-induced sPLA2-IIA expression in BECs. We established that this expression was under the control of Krüppel-Like Factor 2 (KLF2), a particular transcription factor induced by bacterial toxins [65]. KLF2 belongs to the SP-1 zinc-finger transcription factor [66]. Although the signaling pathways by which ExoS induces KLF2 expression are still unknown, our studies showed that KLF2 induction by ExoS is independent of RhoA, one of the potential targets suggested by others [67]. ExoS is a bifunctional toxin with two enzymatic domains, GAP and ADPRT [17], but only the GAP domain is responsible for RhoA inactivation [68]. We also established that ADPRT, but not the GAP domain, plays a role in ExoS-induced sPLA2-IIA synthesis by BECs from CF patients [23].

These findings led us to investigate whether ExoS was able to induce sPLA2-IIA expression in cells other than epithelial cells. Using the T3SS-deficient PAK strains (ΔpscF mutant), we investigated the role of this toxin in gpAMs. Our results showed that this mutant induced sPLA2-IIA expression at similar levels compared to the parental stain. This suggests that, unlike human BECs, ExoS does not affect sPLA2-IIA expression in gpAMs. We also showed that the mutant lacking T2SS induced sPLA2-IIA expression in gpAMs at similar levels compared to the parental stain. These results clearly indicate that neither T2SS nor T3SS is involved in *P*. *aeruginosa*-induced sPLA2-IIA expression in gpAMs (Wu et al., manuscript in preparation).

However, the stimulatory effects of ExoS on sPLA2-IIA contrasted with its inhibitory effects on other mediators of the host immune response, such as cytokines and reactive oxygen species (ROS). Indeed, studies have reported that deletion of the exoS gene resulted in a significant increase in interleukin-8 (IL-8) production by Caco-2 cells. This finding suggested that *P. aeruginosa* produces a serine protease capable of degrading IL-8 in the culture medium of infected Caco-2 cells and that the expression of this protease is inhibited in cells infected with the ΔexoS mutant [69]. More recent studies have shown that *P. aeruginosa* can inhibit the ROS burst in neutrophils and that this inhibition occurs, at least in part, through an ExoS-mediated ADP-ribosylation of Ras in neutrophils [70]. However, the pathophysiological consequences of the opposing effects of ExoS on sPLA2-IIA and other mediators of the innate immune response remained to be elucidated.

### 2.4. Induction of sPLA2-IIA by Porphyromonas Gingivalis and Relevance to Periodontal Disease

*P. gingivalis* is a Gram-negative bacterium known to play a key role in the development of periodontal disease [71]. This bacterium has been shown to induce dramatic expression of sPLA2-IIA in human oral epithelial cells, suggesting that sPLA2-IIA might play a role in periodontal disease [72]. The induction of sPLA2-IIA expression by *P. gingivalis* occurs via a mechanism dependent on TLR activation or T3SS and was initiated by the production of arginine-gingipains (rgpA and rgpB). The latter belongs to the cysteine proteases group unique to this bacterium [72,73] that target a specific sequence of the Notch-1 extracellular domain. This process stimulates the translocation of the Notch-1 intracellular domain into the nucleus, leading to the transcriptional activation of the sPLA2-IIA gene [72].

### 2.5. PGN Is the Main Inducer of sPLA2-IIA Expression by Gram-Positive Bacteria

Our studies using gpAMs as a cell model showed that the Gram-positive bacterium *B. anthracis* can stimulate sPLA2-IIA expression. These studies demonstrated that the cell wall PGN purified from *B. anthracis* induced sPLA2-IIA expression through a process depending on NF-κB activation [26]. However, it is still unclear whether PGN stimulated sPLA2-IIA expression via two PGN recognition proteins, TLR2 or NOD-2 [74]. Recent reports showed that NOD may be involved in cell activation by spores of this bacterium [75]. However, we cannot rule out the fact that other bacterial components of the cell wall or secreted by *B. anthracis* are also involved in sPLA2-IIA expression in gpAMs.

In a subsequent study [28], we attempted to identify the *S. aureus* PAMP(s) and associated receptor(s) that induce sPLA2-IIA expression in gpAMs using PGN purified from *S. aureus* (ligand of TLR2 and NOD2) and LTA (ligand of TLR2). Our results revealed that PGN stimulated sPLA2-IIA expression in gpAMs, whereas LTA had only a limited effect on this expression. This suggested the possible involvement of the NOD2 receptor in PGN-induced sPLA2-IIA expression in gpAMPs [28]. To further verify the role of NOD2 in this process, gpAMs were incubated with the minimal active portions of the PGN, motif MDP (ligand of NOD2), MDPc (inactivated MDP), MALP-2 (ligand of TLR2/6), Pam_3_CSK_4_ (ligand of TLR2/1) or the cytosine guanosine dinucleotide (CpG, agonist of TLR9). Remarkably, only MDP was shown to induce sPLA2-IIA expression by gpAMs. These results indicate that NOD2 is probably the only receptor involved in PGN-induced sPLA2-IIA expression in gpAMs [28].

## 3. Bacterial Toxins That Downregulate sPLA2-IIA Expression

### 3.1. Inhibition of sPLA2-IIA Expression by Bacillus Anthracis Toxins

The exact mechanisms by which *B. anthracis* initiates the anthrax disease are not fully established. However, it is clear that this bacterium multiplicates rapidly in the blood stream, ultimately leading to the death of the host [76]. *B. anthracis* can subvert the host immune response [77,78] via a process involving the action of the *B. anthracis* edema toxin (ET) and lethal toxin (LT). This bacterium releases a binary A-B toxin composed of a single B transporter called a protective antigen (PA) and two alternative A components, lethal factor (LF) or edema factor (EF) [76]. LF and EF act in pairs, with PA leading to LT (=PA + LF) and ET (=PA + EF), respectively. Thus, PA serves as a transporter that delivers LF and EF into the host cell cytosol where they target specific molecular components [76].

#### 3.1.1. Lethal Toxin Downregulates sPLA2-IIA Expression by AMs

Our previous studies showed that the expression of sPLA2-IIA was inhibited by LT during infection with gpAMPs by *B. anthracis* [53]. Next, we investigated the mechanisms by which LT alters sPLA2-IIA synthesis in this cell model. LT exhibits metallo-proteolytic activity toward the N-terminus of the MAPK-kinases. We have shown that this toxin inhibits the phosphorylation of MAPK p38 in gpAMs as well as sPLA2-IIA promoter activity in CHO cells [79]. The inhibition of sPLA2-IIA promoter activity was mimicked by co-transfection with the dominant negative construct of p38 (DN-p38) and reversed by the active form of p38-MAPK (AC-p38) [79]. Both LT and DN-p38 decreased NF-κB luciferase activity. However, neither LT nor a specific p-38 inhibitor interfered with LPS-induced IκBα degradation or NF-κB nuclear translocation in AMs [79]. Therefore, we concluded that sPLA2-IIA expression is induced via sequential MAPK-NF-κB activation and that LT alters this expression by interfering with NF-κB transactivation in the nucleus. This hypothesis is consistent with previous studies suggesting that the regulation of sPLA_2_-IIA in Jurkat cells involves epigenetic silencing by DNA hypermethylation [80].

#### 3.1.2. Edema Toxin Impairs sPLA2-IIA Expression by AMs

In a subsequent study, we showed that ET inhibited sPLA2-IIA expression in gpAMs at the transcriptional level through a cAMP/protein kinase A-dependent process [26]. Moreover, the ET-deficient strains induced sPLA2-IIA expression, whereas the live *B. anthracis* strains expressing the functional ET inhibited this expression [26]. This suggests that *B. anthracis* can induce sPLA2-IIA expression probably via PGN and that ET injected into gpAMPs reduces this expression. This inhibition seems to occur via a cAMP/protein kinase A-dependent mechanism [26]. Our reports revealed that the cAMP increase was transient, reaching basal values within 24 h, in contrast to previous studies showing that cAMP accumulation increased within 48 h or more following ET addition to NIH/3T3 fibroblasts and RAW 267 macrophages [81].

Regardless of the kinetics of the cAMP increase during infection of gpAMPs by *B. anthracis*, the final concentrations of sPLA2-IIA should be considered as a balance between the inducing effect of PGN and the inhibitory effect of ET. However, it is worth highlighting that the effects of cAMP on sPLA2-IIA expression depend on the cell type considered. For example, in contrast to our results with gpAMs, other studies have shown that cAMP stimulates the transcriptional activity of the sPLA2-IIA gene in rat vascular smooth muscle cells. This stimulation depends on the interplay of the CCAAT/enhancer binding protein (C/EBP), NF-κB and Ets transcription factors [82].

Other studies have examined the effects of *B. anthracis* toxins on human dendritic cells and showed that both ET and LT inhibited the production of proinflammatory chemokines by these cells, with LT exhibiting the higher inhibitory effect. However, only LT impaired neutrophil recruitment in this model [83]. The authors suggested that ET and LT act in concert to suppress chemokine expression by human dendritic cells and that this action leads to an alteration in immune cell recruitment.

Taken together, these reports suggest that ET and LT may interfere with the immune system by altering sPLA2-IIA expression or by impairing chemokine expression and neutrophil recruitment, which may play a role in the innate host response to *B. anthracis* infection.

### 3.2. Inhibition of sPLA2-IIA Expression by a Bordetella Pertussis AC-Hly Toxin

The ability to inhibit sPLA2-IIA expression in gpAMs seems to be a general mechanism shared by several bacteria. Indeed, we have shown that a toxin secreted by *Bordetella pertussis* named adenylate cyclasehemolysin (AC-Hly) was also able to impair LPS-induced sPLA2-IIA expression by gpAMs. This inhibition is likely due to an increase in cAMP levels by AC-Hly, which is known to display calmodulin-dependent adenylate cyclase activity [84]. It should be noted, however, that the cyclic AMP-elevating capacity of AC-Hly is sufficient to ensure lung infection but not to ensure full virulence of *B. pertussis* [85]. Additionally, AC-Hly did not affect IL-8 production by gpAMs, suggesting that sPLA2-IIA suppression results from a selective effect of this toxin on sPLA2-IIA transcriptional activity. This finding is consistent with our previous studies showing that *B. anthracis* ET can downregulate sPLA2-IIA expression without affecting IL-8 secretion [84].

### 3.3. S. aureus Adenosine Inhibits sPLA2-IIA Expression and Associated Airway Killing

*S. aureus* produces several molecules, including adenosine, that dampen the host immunity [86]. Adenosine is produced by the highly conserved cell wall-anchored adenosine synthase A (AdsA) via the degradation of ATP, ADP and AMP [87]. We showed that an adenosine-deficient *S. aureus* mutant (∆adsA strain) enhanced the pulmonary expression of sPLA2-IIA in a guinea pig model of lung infection and was cleared more efficiently in the airways compared to the wild-type strain [28]. In addition, the ∆adsA strain induced sPLA2-IIA expression by gpAMs after the phagocytosis of *S. aureus* via a NOD2-NF-κB-dependent mechanism. The addition of exogenous adenosine to cultured gpAMs reduced *S. aureus* phagocytosis by these cells and impaired sPLA2-IIA synthesis. This occurred through a downregulation of p38 phosphorylation via adenosine receptors A2a-, A2b- and associated protein kinase A activation. In addition to its effects on phagocytosis, adenosine also acts as an anti-inflammatory mediator in macrophages via the cAMP/PKA axis [88]. This effect is mediated by altering NF-κB activity through PKA [89].

Taken together, these studies indicate that in the airway, *S. aureus* can escape sPLA2-IIA-mediated killing via adenosine-mediated alteration of phagocytosis and sPLA2-IIA synthesis. These processes also highlight the contribution of NOD2 in modulating sPLA2-IIA expression and suggest a mechanism by which *S. aureus* subverts host immunity based on the inhibition of the production of this antimicrobial enzyme.

## 4. Conclusions

**Figure 2:**
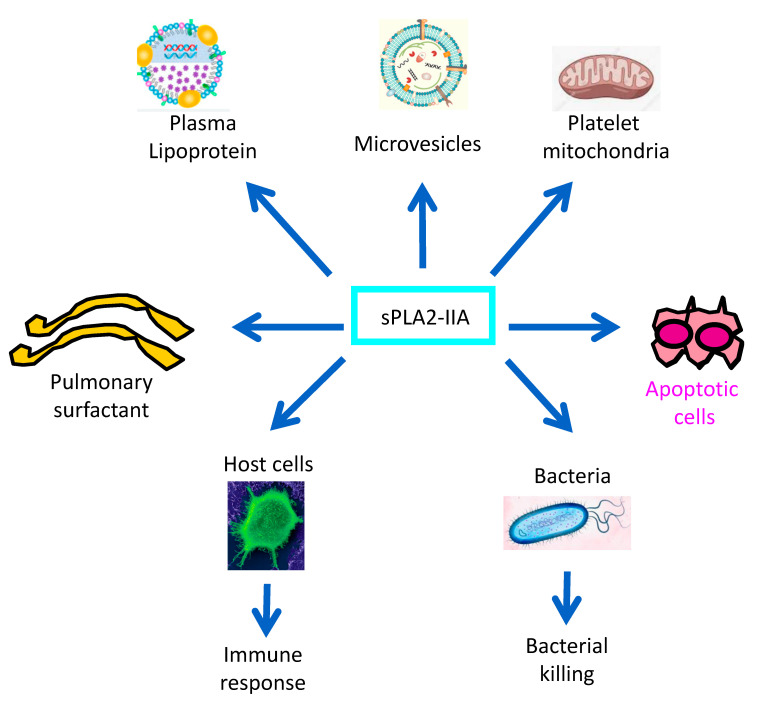
Examples of established biological functions of sPLA2-IIA. sPLA2-IIA targets phospholipids of plasma lipoproteins, microvesicles or mitochondria released by platelets or erythrocytes, thus contributing to the inflammatory processes associated with diseases such as atherosclerosis and sepsis. This enzyme can also hydrolyze pulmonary surfactant phospholipids, an important feature of ARDS pathogenesis. sPLA2-IIA has been also shown to promote neurodegeneration through activation of neuronal cell apoptosis. But, the most studied functions of sPLA2-IIA are related to its bactericidal action and ability to release lipid mediators through the hydrolysis of host cell phospholipids.

As a mediator of the inflammatory response, sPLA2-IIA appears to act as a double-edged sword for the host (Figure 2). Indeed, in addition to the bactericidal functions and their regulation summarized in this review, sPLA2-IIA has been shown to play a role as a proinflammatory mediator involved in pathogenic processes. This includes alteration of pulmonary surfactant, hydrolysis of mitochondrial membrane released by platelets and interaction with plasma lipoproteins. sPLA2-IIA has also been shown to generate lipid mediators from membrane vesicles of platelets and erythrocytes and to play a role in neurotoxicity and cell apoptosis. The bactericidal activity of sPLA2-IIA has emerged as a central function of this enzyme, leading to the elimination of invading pathogenic bacteria and the modulation of microbiota composition. However, further work is required to identify the precise mechanisms involved in the crosstalk between sPLA2-IIA-mediated effects on gut and airway microbiota as well as on lung immune responses.

In general, Gram-negative bacteria, such as *P. aeruginosa*, are insensitive to the bactericidal effects of sPLA2-IIA but can induce its expression by host cells. This results in the selective elimination of Gram-positive bacteria, such as *S. aureus*, which are highly sensitive to sPLA2-IIA killing, a process that is advantageous for Gram-negative bacteria. However, the Gram-positive bacteria can also inhibit sPLA2-IIA expression, highlighting an evolutionary adaptive mechanism by which these bacteria evade the host’s innate immunity.

As shown in this review, several bacterial toxins can regulate sPLA2-IIA levels by either inducing or inhibiting their expression in host cells. These effects depend on the toxins and the host cell type considered. Thus, the final sPLA2-IIA concentration in host biological fluids can be considered as a balance between the stimulatory and inhibitory effects of these toxins. Our review was focused on bacterial toxins, but it is necessary to investigate whether other respiratory pathogens (viruses and fungi) can also modulate sPLA2-IIA expression in the airways and its potential role in the outcome of the infection. Nevertheless, the beneficial bactericidal functions of sPLA2-IIA highlight a potential therapeutic value of this enzyme in respiratory infectious diseases, especially in the context of the antibiotic resistance crisis.

## Figures and Tables

**Table 1:** Effects of bacterial PAMPs and toxins on sPLA2-IIA expression by host cells.

PAMPs	Bacterium	Host PRRs	Effect on sPLA2-IIA Expression		References
			gpAMs	BECs	
LPS	G−	TLR4 ^a^	Upregulation ^b^	No effect ^c^	^a^ [21], ^b^ [22], ^c^ [23]
Peptidoglycan	G+; G−	NOD1 ^a^, NOD2 ^a^	Upregulation ^b^	/	^a^ [24], [25], ^b^ [26]
Lipoteichoic acid	G+	TLR2 ^a^	No effect ^b^	/	^a^ [27], ^b^ [28]
Flagellin	G−	TLR5 ^a^	No effect ^b^	No effect ^c^	^a^ [29], ^b^ our unpublished data, ^c^ [28]
Pili	G+; G−	CD46, CD48, CD55, etc ^a^	Upregulation ^b^	No effect ^c^	^a^ [30], ^b^ [31], ^c^ [23]
HSP60	G+; G−	TLR2, TLR4 ^a^	/	/	^a^ [32]
CpG DNA	G+, G−	TLR9 ^a^	/	No effect ^b^	^a^ [33], ^b^ [23]
HSL	G−	/	/	No effect ^a^	^a^ [23]
ExoS	G−	/	/	Upregulation ^a^	^a^ [23]
Adenosine	G+	Adenosine receptor ^a^	downregulation ^b^	/	^a^ [34], ^b^ [28]
AC-Hly	G−	CD11b/CD18 integrin ^a^	downregulation ^b^	/	^a^ [35], ^b^ [28]
Edema toxin	G+	CMG2 ^a^, TEM8 ^a^	downregulation ^b^	/	^a^ [36], [37], ^b^ [26]
Lethal toxin	G+	CMG2 ^a^, TEM8 ^a^	downregulation ^b^	/	^a^ [36], [37], ^b^ [26]

/: not tested; gAMs: guinea pig alveolar macrophages; BECs: bronchial epithelial cells. a–c: indicate the corresponding references in the table.

## Data Availability

Not applicable.

## Abbreviations

PAMP	pathogen-associated molecular pattern
PRRs	pattern recognition receptors
PLA2	phospholipase A2
sPLA2-IIA	type-IIA secreted phospholipase A2
cPLA2	cytosolic PLA2
G+ bacterium	Gram-positive bacterium
G- bacterium	Gram-negative bacterium
LTA	lipoteichoic acid
PGN	peptidoglycan
LPS	lipopolysaccharides
dsDNA	double-stranded DNA
ARDS	acute respiratory distress syndrome
BALF	bronchoalveolar lavage fluid
AMs	alveolar macrophages
gpAMs	guinea pig alveolar macrophages
BECs	bronchial epithelial cells
DPPC	dipalmitoyl-phosphatidylcholine
BMVECs	brain microvascular endothelial cells
AA	arachidonic acid
PGD2	prostaglandin D2
LOS	lipooligosaccharides
T3SS	type 3 secretion system
ExoS	exotoxin S
CF	cystic fibrosis
KLF2	Krüppel-Like Factor 2
GAP	GTPase activating protein
ADPRT	ADP ribosyltransferase
MDP	muramyl dipeptide
MALP-2	macrophage-activating lipopeptide 2 kDa
CpG	cytosine guanosine dinucleotide
ET	edema toxin
LT	lethal toxin
EF	edema factor
PA	protective antigen
DN-p38	dominant negative construct of p38
AC-Hly	adenylate cyclasehemolysin
C/EBP	CCAAT/enhancer binding protein
cAMP	cyclic adenosine monophosphate
AdsA	adenosine synthase A
